# Non-suicidal Self-Injury in Eating Disordered Patients: Associations with Heart Rate Variability and State-Trait Anxiety

**DOI:** 10.3389/fpsyg.2017.01163

**Published:** 2017-07-07

**Authors:** Cristina Giner-Bartolome, Núria Mallorquí-Bagué, Iris Tolosa-Sola, Trevor Steward, Susana Jimenez-Murcia, Roser Granero, Fernando Fernandez-Aranda

**Affiliations:** ^1^Department of Psychiatry, Bellvitge University Hospital-Bellvitge Biomedical Research Institute (IDIBELL)Barcelona, Spain; ^2^CIBER Fisiopatología Obesidad y Nutrición, Instituto de Salud Carlos IIIBarcelona, Spain; ^3^Department of Clinical Sciences, School of Medicine, University of BarcelonaBarcelona, Spain; ^4^Departament de Psicobiologia i Metodologia, Universitat Autònoma de BarcelonaBarcelona, Spain

**Keywords:** eating disorders, non-suicidal self-injury, anxiety, state-anxiety, trait-anxiety, heart rate variability

## Abstract

**Background:** Non-suicidal self-injury (NSSI) is commonly present in individuals with eating disorders (EDs) and is often employed as a maladaptive emotion regulation strategy to avoid or abate negative emotions. One of the most prevalent negative emotions experienced by self-injurers is anxiety; however, this emotion has not been extensively studied in this population. Thus, the aim of our study was to investigate the influence of anxiety on NSSI in patients with ED from two different dimensions: state anxiety and trait anxiety.

**Methods:** The study comprised a total of 66 females: 12 ED patients with NSSI, 32 ED patients without a history of NSSI, and 22 healthy controls. State and trait anxiety were assessed by means of State-Trait Anxiety Inventory (STAI-S-T) and physiological data [i.e., heart rate variability (HRV)] were collected.

**Results:** STAI-trait scores were significantly higher in ED patients with NSSI than ED patients without NSSI. Furthermore, when conducting logistic regression analyses higher STAI-trait scores were associated with NSSI in ED patients. However, no differences in STAI-state scores and HRV were found between ED patients with and without NSSI.

**Discussion:** The present findings suggest that anxiety as a trait is associated with the use of maladaptive strategies (i.e., NSSI) in ED patients. These results uphold the need to target trait anxiety in ED treatment in order to prevent possible NSSI behaviors.

## Introduction

There is growing evidence indicating that patients with eating disorders (EDs) present difficulties expressing and regulating emotions (Giner-Bartolomé et al., 2016). Increasing evidence supports preceding emotion regulation (ER) models (Linehan, 1993; Gross, 1998) that postulate that ER difficulties often lead to the implementation of less appropriate strategies (e.g., maladaptive food intake or deliberate self-harm) to mollify unpleasant emotions. Likewise, deficits in ER observed among individuals suffering from EDs have been hypothesized to underlie the high prevalence of non-suicidal self-injury (NSSI) behaviors, which range from 21 to 59% in this clinical population (Kostro et al., 2014; Claes et al., 2015b; Islam et al., 2015; Cucchi et al., 2016). Still, among individuals with clinical EDs, those who engage in NSSI have higher dietary restrictions and higher rates of binging and/or purging symptomatology (Stein et al., 2004; Selby et al., 2010).

NSSI behaviors are described as deliberate self-induced harm actions on the surface of one’s body that usually cause bleeding, bruising, or pain (e.g., cutting, burning, and hitting) without suicidal intention (APA, 2013). Notably, similar ER difficulties have been postulated for EDs and NSSI behaviors; namely, both reveal difficulties in thinking, planning, or implementing adaptive coping strategies (Solano et al., 2005; Claes et al., 2010; Muehlenkamp et al., 2012; Vansteelandt et al., 2013; Claes and Muehlenkamp, 2014; Kostro et al., 2014; Islam et al., 2015; Cucchi et al., 2016). Within this clinical population both maladaptive food intake and NSSI behaviors can be implemented as a maladaptive ER strategy that enables the individual to avoid, manage or lessen unpleasant emotions (Chapman et al., 2006; Andover and Morris, 2014; McKenzie and Gross, 2014). In order to better explain this phenomenon, Chapman et al. (2006) proposed the Experiential Avoidance Model (EAM) of NSSI. According to the EAM, NSSI behaviors aim to reduce or eliminate unwanted emotional responses (mostly perceived physiological arousal) and thus, these behaviors are primarily maintained by negative reinforcement.

In physiological terms, emotions can be framed in a body–mind interaction model where both current body state (e.g., physiological arousal) and associated cognitions are coupled to embody the emotional state and subjective experience of the emotion, which in turn can lead to the resulting ER processes. A well-accepted way to assess autonomic nervous system state is through measuring the heart rate variability (HRV) index (Weinberg et al., 2009; Mazurak et al., 2011), a variation in the heart’s beat-to-beat interval (Scolnick et al., 2014; Ramírez et al., 2015). It has been suggested that both higher and lower HRV are mainly determined by the parasympathetic system (Malik et al., 1996; Houle and Billman, 1999; Goldstein et al., 2011; Reyes del Paso et al., 2013; Ramírez et al., 2015). EDs have been associated with alterations in the autonomic nervous system, however, results are still inconclusive with most studies accounting increased HRV to parasympathetic nervous system dominance, whereas others describe decreased HRV conveying dominance of the sympathetic nervous system, or no differences comparing HRV in ED patients to controls (Mazurak et al., 2011; Peschel et al., 2016a,b). Additionally, HRV is an objective measure that has been considered as an index of ER capacity in a large number of studies (Di Simplicio et al., 2012; Thayer et al., 2012; Tuck et al., 2016; Visted et al., 2017). Specifically, it has been proposed that higher HRV is associated with a greater capacity to regulate negative emotions in terms of emotional clarity (i.e., the capacity to identify these emotions) and emotional impulse-control (to adaptively inhibit or regulate emotional responses). Likewise, lower HRV is associated with the ER difficulties observed in certain psychopathological vulnerabilities, such as trait anxiety (a relatively stable, temperamental predisposition to anxiety; Williams et al., 2015). Also, decreased HRV is observed in anxious states (i.e., transitory emotional state characterized by subjective feelings of attention, apprehension and autonomic nervous system hyperactivity), which are associated with increased physiological effects [increased heart rate (HR)], sweating and decreased HRV (Rodas et al., 2008; Kreibig, 2010; Chevalier and Sinatra, 2011) and vagal deactivation. Although most studies measuring HRV are based on resting paradigms, some studies explore HRV as a biofeedback measure (Siepmann et al., 2008; Lehrer and Gevirtz, 2014). To date, there are limited studies on the use of biofeedback in the treatment of ED (Meule et al., 2012; Fagundo et al., 2013). Still, it could be of interest to see how individuals differ in in-task HRV during biofeedback sessions.

Importantly, anxiety is highly linked to ER capacity (Hu et al., 2014; Greening and Mitchell, 2015; Sakakibara and Kitahara, 2016) and it has been described as one of the main unpleasant emotions commonly experienced by NSSI patients. It is also one of the main factors associated with a greater likelihood of engaging in these behaviors (Fliege et al., 2009; Selby et al., 2012; You et al., 2016). Specifically, Greening and Mitchell (2015) reported that higher trait anxiety was associated with negative ER strategies through stronger connections between the amygdala and dorsal anterior cingulate cortex (areas involved in emotional reactivity) and the inferior temporal gyrus and paracentral lobule (areas associated with perceptual and sensory processing). However, despite its relevant implications on the EDs and NSSI field, no studies have been conducted so far focusing on state/trait anxiety and HRV indices with in this population.

As such, the main aims of the present study were the following: (1) to compare state, trait anxiety and in-task HRV (measured through a serious videogame in a biofeedback task that triggers the implementation of ER strategies) in three different groups: ED with NSSI (ED+NSSI), ED without NSSI (ED-NSSI), and healthy controls (HC), and (2) to test state-trait anxiety and in-task HRV as independent predictors of NSSI in ED+NSSI patients in a cross-sectional study. In-task HRV indices were obtained while participants were engaged in the Playmancer (Fernandez-Aranda et al., 2015) biofeedback videogame implemented to train the regulation of the elicited unpleasant emotions (e.g., anxiety, frustration). We hypothesize that: (1) ED+NSSI patients will present higher levels of state and trait anxiety than ED-NSSI patients, and that both groups will differ in these measures when compared with HC; (2) State-trait anxiety and in-task HRV will independently predict the presence of NSSI in ED+NSSI patients.

## Materials and Methods

### Participants

A total of 66 participants were included in this study: 22 HC, 32 ED-NSSI, and 12 ED+NSSI. The ED-NSSI group was 59% bulimia nervosa (BN, *n* = 19), 25% anorexia nervosa restrictive type (AN-R, *n* = 18), 12.5% anorexia nervosa binge-eating/purging type (AN-B/P, *n* = 4) and 3% of otherwise specified feeding or ED purging subtype (OSFED-P, *n* = 1). The ED+NSSI group was 50% BN (*n* = 6), 25% AN-R (*n* = 3), 17% binge ED (BED, *n* = 2) and 8% AN-B/P (*n* = 1). The prevalence of NSSI among the different ED diagnoses of our total sample was 100% in BED, 24% in BN, 20% in AN-B/P, 14% in AN-R and 0% in OSFED-P. The presence of ED were diagnosed by experienced psychologists and psychiatrists according to DSM-IV-TR criteria (APA, 2000) by means of a semi-structured clinical interview adapted from the SCID-I (First et al., 1997). ED diagnoses were recoded *post hoc* according to DSM-5 diagnostic criteria (APA, 2013). ED patients were consecutive referrals for assessment and treatment at the Psychiatry Department of the University Hospital of Bellvitge (Barcelona, Spain). Only women ranging from 18 to 60 years of age were included in the study sample. All patients were classified in two groups according to presence of lifetime history of NSSI behaviors or absence of lifetime history of NSSI behaviors. The HC group was made up of volunteer participants from our hospital staff and student interns. Exclusion criteria for the whole sample at intake were as follows: having a primary psychiatric or neurological disorder (psychotic disorder, bipolar disorder, major depressive disorder, substance abuse and/or dependence, or epilepsy), and/or receiving a pharmacological treatment that may interfere with the recording of physiological data. In the ED-NSSI group, patients could not have a history of NSSI behaviors. HC did not have a history of ED, NSSI behaviors, or any other psychopathological conditions.

This study was carried out in accordance with the recommendations of the Ethics Committee of the University Hospital of Bellvitge with written informed consent being obtained from all subjects in accordance with the Declaration of Helsinki.

### Assessment

#### Evaluation of NSSI Behaviors and Other Clinical Variables

At the beginning of the study, a clinical interview was conducted to explore questions such as ED symptoms using the SCID-I (First et al., 1997) and the presence of associated impulsive behaviors (such as drug or alcohol abuse/dependence). Psychometric and anthropometric measures [such as body mass index (BMI)] were also taken. Similar to Islam et al. (2015), the existence of current or lifetime regular NSSI behaviors was assessed by means of a clinical interview by experienced psychologist and psychiatrists. We considered NSSI behaviors to be self-cutting, burning, hitting, and scratching, not performed with a suicidal intent. Drug or alcohol abuse, bingeing and vomiting behaviors were not included as NSSI.

#### Psychometric Measures

*Symptom Checklist revised* (*SCL-90-R*; Derogatis, 1990, 2002): This 90-item questionnaire is widely used for the measurement of self-reported overall psychological distress and psychopathology. It is scored with the following dimensions: Somatization, Obsessive–Compulsive, Interpersonal Sensitivity, Depression, Anxiety, Hostility, Phobic Anxiety, Paranoid Ideation, and Psychoticism. A global severity index is used as a global distress index. The Spanish validation of this instrument has shown a mean internal consistency of α = 0.75 (Martinez-Azumendi et al., 2001). Given that depression has been shown to influence the occurrence of NSSI (Fliege et al., 2009; Selby et al., 2012; You et al., 2016), the depression subscale was used in this study as a covariate. All the subscales have good internal consistency in this study (α ranged from 0.822 to 0.982).

*State-Trait Anxiety Inventory* (*STAI-S-T*; Spielberger et al., 1970): This is a self-report questionnaire that includes 40 items. It consists of two subscales, each one made up of 20 items, that measure “state anxiety” and “trait anxiety.” The Spanish adaptation (Spielberger et al., 1982) obtained good reliability indices in psychometric studies. Cronbach’s alpha reliability is 0.90 for trait anxiety and 0.94 for state anxiety (Guillen-Riquelme and Buela-Casal, 2011). In our sample, Cronbach’s alpha reliability is 0.940 for trait anxiety and 0.961 for state anxiety.

*Eating Disorders Inventory-2* (*EDI-2*; Garner, 1991): This 91-item questionnaire assesses cognitive and behavioral characteristics that are common in ED. It contains eight scales (Drive for Thinness, Bulimia, Body Dissatisfaction, Ineffectiveness, Perfectionism, Interpersonal Distrust, Interoceptive Awareness, and Maturity Fears) and three provisional scales (Asceticism, Impulse Regulation, and Social Insecurity) answered on a 6-point Likert scale. In the present study, the EDI-2 total score, understood as a severity grade, was used in statistical analyses to compare each group’s characteristics. It has been validated in a Spanish population (Garner, 1998) with a mean internal consistency of 0.63 (coefficient alpha). Cronbach’s alpha reliability of the EDI-2 total scores of this sample is 0.958.

#### Physiological Measure

The physiological variable employed in this study was in-task HRV. This measure was recorded while participants played a serious videogame named “Playmancer.” Serious games are games designed for a specific purpose that goes beyond that of pure entertainment. They are applied with an explicit goal in contexts such as healthcare, with educational, preventive, and/or therapeutic purposes (Gaudet-Blavignac and Geissbuhler, 2012). As described in previous studies (Jimenez-Murcia et al., 2009; Fernandez-Aranda et al., 2012), our serious game is based on biofeedback techniques and is used as an additional therapeutic tool, combined with standard psychological treatment approaches for ED. The main goal of this intervention is to teach strategies to enhance self-control and to regulate negative emotions (e.g., anxiety and frustration) when the individual has to face different challenges. Playmancer treatment usually lasts 10 weeks with a session per week. The challenges are presented via three different activities (mini-games) that the player has to carry out: (1) *The Face of Cronos*: In this game, the player must reach the top of a cliff, trying to dodge different obstacles that appear during the climb according to the individual’s arousal level (higher physiological activation leads to the appearance of more obstacles); (2) *Treasures of the Sea*: In this swimming game, the player must find different hidden treasures while trying to regulate their oxygen consumption. Higher physiological activation causes an increase in the oxygen consumption, making the task more difficult to complete; and (3) *Sign of the Magupta*: In this relaxation game, the player has to complete different constellations of stars via slow, deep breathing techniques.

**FIGURE 1:**
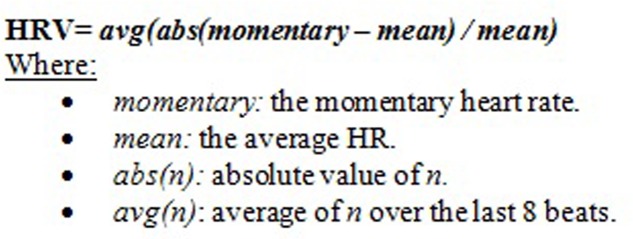
Hart rate variability (HRV) measurement formula.

As a part of our videogame system and for biofeedback purposes, electrocardiogram (ECG) sensors are placed to measure the participants’ electrical heart activity. To do so, two skin electrodes are placed on the chest, as follows: one electrode (negative) is placed above the ninth-tenth rib, on the right side; the other electrode (positive) is also placed above the 9th to 10th rib, but on the left side. These sensors are the source for ECG lead, HR, and HRV. Thereby, from other biofeedback variables, this videogame program calculates the average HRV of the player during the session. HRV reflects the fluctuation in HR. In our videogame system, HRV is given as percentage relative to the average HR. If the last two beats found by the beat-to-beat algorithm are at times *T*_n__-1_ and *T*_n_ (in seconds), then the momentary HR is (60/Δ*T*) where Δ*T* = *T*_n_ -*T*_n__-1_. HRV is the difference between momentary and average HR, averaged over the last eight beats to reduce the impact of breathing (**Figure 1**). HRV was acquired through the TMSi Mobi8 amplifier sensor system. Sensors are connected via wires to the Mobi8. The Mobi8 receives measurement data from sensors and sends them via Bluetooth^TM^ to the Playmancer desktop PC application where data is recorded. Each recording session lasts 26 min. Higher HRV reflects less physiological activation, whereas lower HRV indicates more physiological activation and, thus, increased anxiety levels (Malik et al., 1996; Scolnick et al., 2014; Ramírez et al., 2015). For more information about this serious game, see Jimenez-Murcia et al. (2009) and Fernandez-Aranda et al. (2012).

### Procedure

All participants with EDs (ED+NSSI and ED-NSSI) were assessed with a clinical interview and completed the aforementioned questionnaires, as part of the assessment and diagnostic protocol of our Unit. All anxiety measures were completed within the same day, including questionnaires and the ER biofeedback task (Playmancer serious videogame). HC participants followed the same procedure: they first completed the diagnostic protocol and self-reported questionnaires followed by the Playmancer task. Although Playmancer treatment lasts 10 sessions (see Fernandez-Aranda et al., 2015 for further description), in this study, we used the information from the first session, since we aimed to measure “baseline” in-task HRV from all participants, that is, when they have not yet modified any of their physiological patterns as a product of the ER training.

### Statistical Analysis

Statistical analysis was carried out with STATA13.1 for Windows. Firstly, analysis of variance (ANOVA) adjusted for the covariates SCL-90R depression score and ED subtype compared the means for clinical variables and anxiety dimensions between the three independent groups (HC, ED-NSSI, and ED+NSSI). Regarding using the SCL-90R depression subscale as a covariable, it was included since previous studies have described that depressive state is a powerful risk factor for the presence of NSSI (Fliege et al., 2009); therefore, the inclusion of depressive symptom levels avoid the potential bias due to its confounding effects. The ANOVA analyses included pairwise comparisons through Bonferroni’s method and estimated the mean difference size through Cohen’s-*d* coefficient (|*d*| > 0.50 was considered moderate and |*d*| > 0.80 was considered high).

Secondly, Pearson’s correlations described the relationships between the main variables of the study. The correlation matrix was stratified by diagnostic subtype (coefficients were separately estimated for the HC, ED-NSSI, and ED+NSSI subsamples) since the associations between the variables could be different depending on the diagnostic group. This correlation matrix was included with two main objectives: (a) to describe the pattern of relationships for the main variables of the study, and (b) to identify potential problems due to the presence of collinearity between variables that could affect the predictive logistic models.

Thirdly, a logistic regression assessed the predictive capacity of STAI scales (state and trait) and HRV for the presence of the NSSI behavior (0 = absent versus 1 = present) in the ED samples. This model was programmed in two blocks/steps: (a) the first block included and set the covariates: patient’s age, BMI (since it has been reported that HRV is related to BMI; Koenig et al., 2014, 2015), SCL-90R depression and the ED subtype; and (b) the second block added anxiety and HRV measures. Goodness-of-fit was measured with the Hosmer–Lemeshow test (*p* > 0.05 was considered adequate fitting), the global predictive capacity with the Nagelkerke’s pseudo-*R*^2^ coefficient (the adjusted contribution of the predictors was calculated as the increase between blocks 1 and 2, Δ*R*^2^) and the global discriminative capacity with the area under the ROC curve (AUC).

## Results

### Comparison of Clinical and Anxiety Measures between Groups

**Table 1** includes the results of the ANOVA (adjusted for SCL-90-R depression and ED subtype) comparing mean scores of clinical variables and anxiety dimensions between the three groups of the study, pairwise comparisons and effect size (Cohen’s-d value). Participants’ age, age of onset of the ED, duration of the ED, and BMI were equally distributed between groups, and only one mean difference in the moderate range was found: the pairwise comparison of the chronological age between the two ED groups (31.8 for ED-NSSI versus 26.8 for EDI+NSSI; *d* = 0.53). Considering anxiety measures, more relevant differences emerged. First, compared to both the ED-NSSI and ED+NSSI groups, HC registered lower mean scores in the STAI-state and STAI-trait scales, but no differences among groups were observed in in-task HRV indices. Second, ED+NSSI obtained higher means compared to the ED-NSSI group on the STAI-trait scale. ED severity (EDI-2 total score) significantly and clinically differed comparing HC with both ED subtypes, while differences between ED-NSSI and ED+NSSI patients were non-significant.

**Table 1:** Comparison of clinical variables and anxiety dimensions between groups.

	HC		ED-NSSI		ED+NSSI		Factor			Pairwise comparisons					
					
	G1; *n* = 22		G2; *n* = 32		G3; *n* = 12		group			G1–G2		G1–G3		G2–G3	
							
	Mean	*SD*	Mean	*SD*	Mean	*SD*	*F*	*df*	*p*	*p*	*|d|*	*p*	*|d|*	*p*	*|d|*
Age (year)	28.55	7.88	31.77	9.70	26.83	8.78	1.65	2/63	0.201	0.199	0.37	0.597	0.21	0.109	**0.53^†^**
Age of onset (year)			21.19	8.02	17.92	8.30	1.43	1/32	0.239					0.239	0.40
Duration ED (year)			10.58	9.05	8.92	6.35	0.34	1/32	0.562					0.562	0.21
BMI (kg/m^2^)	20.81	1.85	21.30	4.74	23.83	9.48	1.06	2/63	0.353	0.800	0.14	0.200	0.44	0.195	0.34
^1^SCL-90R: depression	0.83	0.32	1.81	1.05	2.63	0.89	5.25	2/63	**0.009^∗^**	**0.048^∗^**	**1.16**	**0.013^∗^**	**2.68**	**0.017^∗^**	**0.84^†^**
^1^ED severity: EDI-2 total	44.5	11.2	85.4	45.2	90.2	41.1	9.05	2/63	**0.001^∗^**	**0.001^∗^**	**1.24^†^**	**0.002^∗^**	**1.52^†^**	0.636	0.11
^2^Heart rate variability	4.68	2.09	5.20	2.38	4.25	1.64	0.75	2/63	0.479	0.749	0.23	0.794	0.24	0.229	0.46
^2^STAI: anxiety-state	20.61	10.31	28.35	8.85	31.46	9.97	1.51	2/63	0.230	0.160	**0.81^†^**	0.088	**1.07^†^**	0.354	0.33
^3^STAI: anxiety-trait	18.79	6.95	33.89	9.08	39.43	5.06	8.75	2/63	**0.001^∗^**	**0.001^∗^**	**1.87^†^**	**0.001^∗^**	**3.40^†^**	**0.039^∗^**	**0.75^†^**


*SD, standard deviation; |*d*|, Cohen’s-*d* measuring effect-size; Df, degrees of freedom. HC, healthy controls; ED-NSSI, eating disorders without non-suicidal self-injury; ED+NSSI, eating disorders with non-suicidal self-injury.*

*^1^Adjusted for ED subtype.*

*^2^Adjusted for age, BMI, SCL-90R depression score, and ED subtype.*

*^3^Adjusted for SCL-90R depression score and ED subtype.*

*^∗^Bold: significant comparison (0.05 level).*

*^†^Bold: effect size in the moderate (|*d*| > 0.50) to high range (|*d*| > 0.80).*

### Correlations between the Main Variables of the Study

The Pearson’s correlation-matrix for the main variables of the study, stratified by the diagnostic subtype, describes the pattern of associations in each group. In ED-NSSI patients: (a) the HRV index negatively correlated with the age (*r* = -0.345, *p* < 0.05); (b) the state and trait anxiety scores positively correlated with ED severity (*r* = 0.323, *p* < 0.05 and *r* = 0.540, *p* < 0.001, consecutively), and with depression (*r* = 0.370, *p* < 0.05 and *r* = 0.516, *p* < 0.001); and (c) state and trait anxiety positively inter-correlated (*r* = 0.586, *p* < 0.05). In the ED+NSSI group more associations and with a higher effect size emerged: (a) HRV index positively correlated with ED severity (*r* = 0.256, *p* < 0.05); (b) and state anxiety correlated with age (*r* = 0.686, *p* < 0.05), BMI (*r* = 0.469, *p* < 0.05), ED severity (*r* = 0.310, *p* < 0.05) and depression (*r* = 0.338, *p* < 0.05); and (c) trait anxiety correlated with and ED severity (*r* = 0.267, *p* < 0.05) and HRV (*r* = 0.385, *p* < 0.05). Finally, in HC: (a) HRV index negatively correlated with age (*r* = -0.708, *p* < 0.001) and positively with anxiety trait levels (*r* = 0.304, *p* < 0.05); and (b) anxiety state correlated with depression levels (*r* = 0.315, *p* < 0.05); and (c) anxiety state and trait levels positively correlated (*r* = 0.492, *p* < 0.05).

This correlation matrix did not evidence the presence of very high correlations between variables that could affect the predictive models due to collinearity.

### Predictive Model for the Presence of NSSI in the ED Groups

**Table 2** presents the results of the second block-step for the final logistic regression assessing the predictive capacity of anxiety (STAI scales) and in-task HRV indices for the presence of NSSI. Adjusted for the covariates patient’s age, BMI, SCL-90-R depression and ED subtype, the risk of presenting NSSI was increased for ED patients with higher STAI-trait scores. STAI-state scale scores and in-task HRV indices did not statistically contribute to the outcome. This model achieved adequate fit (*p* = 0.875) and high predictive and discriminative capacity (*R*^2^ = 0.18, AUC = 0.84).

**Table 2:** Logistic regression assessing the predictive capacity of anxiety and in-task HRV for the presence of NSSI in the ED sample.

	*B*	SE	Wald	*p*	OR	95% CI	OR
Constant	-8.208	4.072	4.062	0.044	0.00		
Age (year)	-0.137	0.076	3.213	**0.030^∗^**	0.87	0.75	1.00
BMI (kg/m^2^)	0.168	0.107	2.438	0.080	1.18	0.96	1.46
SCL-90R depression (covariate)	0.554	0.557	0.992	0.297	1.74	0.58	5.18
ED subtype (covariate)	1.418	1.306	1.179	0.261	4.13	0.32	53.33
STAI: anxiety-trait	0.152	0.082	3.457	**0.044^∗^**	1.16	1.00	1.37
STAI: anxiety-state	0.015	0.058	0.066	0.797	1.01	0.91	1.14
HRV index	-0.249	0.253	0.969	0.303	0.78	0.48	1.28
Fitting: H–L: 0.594; Δ*R*^2^ = 0.140; AUC = 0.891							


*ED, eating disorder; H–L, Hosmer–Lemeshow test; Δ*R*^*2*^, increase in the Nagelkerke’s *R*-square after adjusting for the covariate depression; AUC, area under ROC curve.*

*^∗^Bold: significant comparison (0.05 level).*

## Discussion

This study aimed to compare state/trait anxiety and in-task HRV indices (through a serious videogame in a biofeedback task that triggers the implementation of ER strategies) in three different samples: ED patients with NSSI, ED patients without NSSI and HC. We also sought to test state-trait anxiety and in-task HRV as independent predictors of NSSI in ED patients. Results show that ED patients have significantly higher trait anxiety scores and higher state anxiety scores with significant effect sizes, although without significant *p* values, than HC. Still, no significant differences are observed for the in-task HRV indices. Also and most importantly, results reveal that trait anxiety plays a pivotal role in the presence of NSSI behaviors among the ED patients.

Individuals with ED have been widely described as a population with poor ER skills (Harrison et al., 2010; Svaldi et al., 2012; Brockmeyer et al., 2014; Danner et al., 2014) as well as with higher levels of state anxiety (Keski-Rahkonen and Mustelin, 2016) and trait anxiety (Solano Pinto and Cano Vindel, 2012) when compared to HC. Our data adds additional evidence of the higher rates of anxiety among these individuals and stresses the need to target these symptoms for treatment purposes.

Additionally, NSSI behaviors are highly prevalent among patients with disordered eating (Kostro et al., 2014; Claes et al., 2015b; Islam et al., 2015; Cucchi et al., 2016) and both ER difficulties, as well as high anxiety scores, may account for this association (Solano et al., 2005; Muehlenkamp et al., 2012; Vansteelandt et al., 2013). In line with these results, previous studies have also suggested that trait and state anxiety are triggering factors for NSSI behaviors (Selby et al., 2012; You et al., 2016), with trait anxiety described as a predisposing factor for the occurrence of NSSI (Fliege et al., 2009; Salman et al., 2014). Based on this previous literature, we hypothesized that ED patients with NSSI behaviors would also present higher state-trait anxiety scores when compared to ED patients without NSSI behaviors. However, contrary to our hypothesis we did not find any differences in state anxiety between EDs with NSSI and EDs without NSSI. Instead, the results of this study suggest that the NSSI behaviors in EDs are more associated with higher trait anxiety. One explanation for this finding is that, in comparison with previous studies, our ED sample (with and without NSSI) may have presented similar levels of state anxiety mainly due to their shared ED condition. Nonetheless, these results need to be interpreted cautiously and further explored in future studies.

Additionally, our logistic regression model analysis demonstrated that trait anxiety was the only variable that predicts NSSI behaviors among the ED patients in our sample. Considering that individuals with NSSI often use these behaviors as a way to manage negative emotions (Chapman et al., 2006; Andover and Morris, 2014; McKenzie and Gross, 2014), it stands to reason that those who usually feel anxious are more likely to commit NSSI. Thus, the general predisposition to feel anxious, rather than the state experience of anxiety, could be a main factor to consider in the study and for the development of therapeutic approaches for ED patients at risk of NSSI behaviors. From a clinical perspective, special attention should be paid to those patients with ED who present higher trait anxiety levels in order to provide ER interventions and help with the prevention of possible NSSI behaviors.

Finally, EDs have been associated with alterations in the autonomic nervous system (Mazurak et al., 2011; Peschel et al., 2016a,b). In this regard, we found no significant in-task HRV differences when conducting the Playmancer-ER task. Although most previous studies have reported increased in-task HRV (Mazurak et al., 2011), our findings are in line with other studies reporting no differences in HRV reactivity in ED (Peschel et al., 2016b). Also, we explored in-task HRV differences between the ED with and without NSSI. The rational for exploring these differences through the Playmancer-ER task was to observe different physiological indices associated with the presence of NSSI behaviors, which are highly associated with ER difficulties. Previous studies have reported different physiological indices in NSSI compared to those who do not present NSSI (Nock and Mendes, 2008), however, our results do not support these findings. The reported divergences could possibly be due to different methods for assessing HRV or to constrained sample sizes. Nevertheless, our results raise the necessity to further explore these variables in order to better understand alterations in the autonomic nervous system in ED patients.

To our knowledge, few studies on ED patients with NSSI have exclusively focused on the role of anxiety in itself. In this regard, our study is the first that explores which dimension of anxiety (state or trait) is associated with NSSI behaviors in individuals with ED, including not only subjective (self-reported questionnaires) but also objective (physiological) measurements. Thus, this research contributes to conceptualize NSSI in ED and raises many questions worthy of further investigation.

Nevertheless, the generalization of the reported results is subject to certain limitations. First, based on previous studies (Pisetsky et al., 2017) and given that we were considering ED along continuum with ER problems and disordered eating, this study has not differentiated between different EDs. However, future studies should explore differences between disorders. Also, more studies are needed with larger samples and less sample size differences which also include males, before the obtained results can be generalized. Additionally, recall biases might also be a limiting factor inherent in retrospective and cross-sectional assessments. Thus future longitudinal studies are required to specifically determine the role of state and trait anxiety as predictors of NSSI in ED patients. With regards to the assessment techniques, Playmancer in-task HRV indices are adequate for measuring HRV indices through an ER task (Siepmann et al., 2008; Lehrer and Gevirtz, 2014) but results may be linked to different emotions (e.g., anxiety, frustration) and this study does not provide extra manipulation check measures. Given that previous studies often use the classic baseline and recovery period HRV measures with stress or anxiety provoking tasks, our in-task HRV results, which are based on a biofeedback-ER task are still difficult to compare with the current literature. Moreover, we explored the presence of NSSI only through a clinical interview without including a psychometric assessment, which would have allowed us to obtain more detailed information about subjects’ self-harm behaviors (e.g., frequency, intensity, etc.). We cannot omit the role that other factors such as impulsivity and compulsivity could also have in the occurrence of these NSSI behaviors (Claes et al., 2015a). However, together with impulsivity and compulsivity, trait anxiety should also be considered in future studies in ED NSSI behaviors. Regarding depressive symptoms, it must be highlighted that this factor could also bias the results; though we did control for this variable to minimize its impact. Future studies could also target depressive symptoms and compare them with trait anxiety scores to see if these two factors equally predispose NSSI behaviors in ED patients. Finally, since this study was carried out with a low sample size for the comparisons between groups (statistical power was low), mean differences with significant (*p* < 0.05) or moderate to high effect sizes (|*d*| > 0.50) were considered as relevant in the work.

## Conclusion

In conclusion, our results suggest that trait anxiety plays a larger role than state anxiety in the occurrence of NSSI in patients with ED. Anxious temperament could be a main factor in choosing maladaptive strategies (i.e., NSSI behaviors) as a mechanism to regulate negative emotions. From a clinical perspective, these results uphold that special attention should be paid to those patients with ED who present high trait anxiety levels and that such patients should be provided with targeted ER interventions in order to prevent possible NSSI behaviors. Although the results of the present study are promising, further research through longitudinal studies is needed to corroborate these findings.

## Author Contributions

CG-B, NM-B, IT-S, TS, RG, SJ-M, and FF-A have substantial contributions to the three following processes:

(1) Conception or design of the work: CG-B, NM-B, IT-S, SJ-M, and FF-A.
(2) Acquisition, analysis, or interpretation of data for the work: RG, CG-B, NM-B, IT-S, and FF-A.
(3) Drafting the work or revising it critically for important intellectual content: CG-B, NM-B, IT-S, TS, RG, SJ-M, and FF-A.

All authors express the agreement to be accountable for all aspects of the work in ensuring that questions related to the accuracy or integrity of any part of the work are appropriately investigated and resolved.

## Conflict of Interest Statement

The authors declare that the research was conducted in the absence of any commercial or financial relationships that could be construed as a potential conflict of interest.

## References

[B1] Andover M. S. Morris B. W. (2014). Expanding and clarifying the role of emotion regulation in nonsuicidal self-injury. *Can. J. Psychiatry* 59 569–575. 25565472 10.1177/070674371405901102 PMC4244875

[B2] APA (2000). *DSM-IV-TR: Diagnostic and Statistical Manual of Mental Disorders*, 4th Edn Washington, DC: American Psychiatric Association.

[B3] APA (2013). *Diagnostic and Statistical Manual of Mental Disorders (DSM-5)*, 5th Edn Washington, DC: American Psychiatric Association.

[B4] Brockmeyer T. Skunde M. Wu M. Bresslein E. Rudofsky G. Herzog W. (2014). Difficulties in emotion regulation across the spectrum of eating disorders. *Compr. Psychiatry* 55 565–571. 10.1016/j.comppsych.2013.12.001 24411653

[B5] Chapman A. L. Gratz K. L. Brown M. Z. (2006). Solving the puzzle of deliberate self-harm: the experiential avoidance model. *Behav. Res. Ther.* 44 371–394. 10.1016/j.brat.2005.03.005 16446150

[B6] Chevalier G. Sinatra S. T. (2011). Emotional stress, heart rate variability, grounding, and improved autonomic tone: clinical applications. *Integr. Med.* 10 16–21.

[B7] Claes L. Fagundo A. B. Jiménez-Murcia S. Agüera Z. Giner-Bartolome C. Granero R. (2015a). Is non-suicidal self-injury related to impulsivity in anorexia nervosa? Results from self-report and performance-based tasks. *Eur. Eat. Disord. Rev.* 23 28–33. 10.1002/erv.2329 25331260

[B8] Claes L. Klonsky E. D. Muehlenkamp J. Kuppens P. Vandereycken W. (2010). The affect-regulation function of nonsuicidal self-injury in eating-disordered patients: which affect states are regulated? *Compr. Psychiatry* 51 386–392. 10.1016/j.comppsych.2009.09.001 20579512

[B9] Claes L. Luyckx K. Bijttebier P. Turner B. Ghandi A. Smets J. (2015b). Non-suicidal self-injury in patients with eating disorder: associations with identity formation above and beyond anxiety and depression. *Eur. Eat. Disord. Rev.* 23 119–125. 10.1002/erv.2341 25504562

[B10] Claes L. Muehlenkamp J. (2014). “Non-suicidal self-injury and eating disorders: dimensions of self-harm,” in *Non-Suicidal Self-Injury in Eating Disorders*, eds Claes L. Muehlenkamp J. (Berlin: Springer), 3–18. 10.1007/978-3-642-40107-7

[B11] Cucchi A. Ryan D. Konstantakopoulos G. Stroumpa S. Kaçar A. Ş. Renshaw S. (2016). Lifetime prevalence of non-suicidal self-injury in patients with eating disorders: a systematic review and meta-analysis. *Psychol. Med.* 46 1345–1358. 10.1017/S0033291716000027 26954514

[B12] Danner U. N. Sternheim L. Evers C. (2014). The importance of distinguishing between the different eating disorders (sub)types when assessing emotion regulation strategies. *Psychiatry Res.* 215 727–732. 10.1016/j.psychres.2014.01.005 24491687

[B13] Derogatis L. R. (1990). *SCL-90-R. Administration, Scoring and Procedures Manual*. Baltimore, MD: Clinical Psychometric Research.

[B14] Derogatis L. R. (2002). *SCL-90-R. Cuestionario de 90 Síntomas-Manual*. Madrid: TEA Editorial.

[B15] Di Simplicio M. Costoloni G. Western D. Hanson B. Taggart P. Harmer C. J. (2012). Decreased heart rate variability during emotion regulation in subjects at risk for psychopathology. *Psychol. Med.* 42 1775–1783. 10.1017/S0033291711002479 22067596

[B16] Fagundo A. B. Santamaría J. J. Forcano L. Giner-Bartolomé C. Jiménez-Murcia S. Sánchez I. (2013). Video game therapy for emotional regulation and impulsivity control in a series of treated cases with Bulimia Nervosa. *Eur. Eat. Disord. Rev.* 21 493–499. 10.1002/erv.2259 24092598

[B17] Fernandez-Aranda F. Jimenez-Murcia S. Santamaría J. J. Giner-Bartolomé C. Mestre-Bach G. Granero R. (2015). The use of videogames as complementary therapeutic tool for cognitive behavioral therapy in Bulimia Nervosa patients. *Cyberpsychol. Behav. Soc. Netw.* 18 744–751. 10.1089/cyber.2015.0265 26583754

[B18] Fernandez-Aranda F. Jimenez-Murcia S. Santamaria J. J. Gunnard K. Soto A. Kalapanidas E. (2012). Video games as a complementary therapy tool in mental disorders: playMancer, a European multicentre study. *J. Ment. Health* 21 364–374. 10.3109/09638237.2012.664302 22548300 PMC3433177

[B19] First M. B. Spitzer R. L. Gibbon M. Williams J. B. W. (1997). *Structured Clinical Interview for DSM-IV Axis I Disorders-Clinical Version (SCID-CV)*. Washington, DC: American Psychiatric Press.

[B20] Fliege H. Lee J.-R. Grimm A. Klapp B. F. (2009). Risk factors and correlates of deliberate self-harm behavior: a systematic review. *J. Psychosom. Res.* 66 477–493. 10.1016/j.jpsychores.2008.10.013 19446707

[B21] Garner D. M. (1991). *Eating Disorder Inventory-2.* Odessa: Psychological Assessment Resources.

[B22] Garner D. M. (1998). *Inventario de Trastornos de la Conducta Alimentaria (EDI-2)-Manual.* Madrid: TEA.

[B23] Gaudet-Blavignac C. Geissbuhler A. (2012). Serious games in health care: a survey. *Yearb. Med. Inform.* 7 30–33. 22890338

[B24] Giner-Bartolomé C. Steward T. Wolz I. Jiménez-Murcia S. Granero R. Tárrega S. (2016). The influence of personality traits on emotion expression in bulimic spectrum disorders: a pilot study. *Eur. Eat. Disord. Rev.* 24 320–328. 10.1002/erv.2446 27028106

[B25] Goldstein D. S. Bentho O. Park M.-Y. Sharabi Y. (2011). Low-frequency power of heart rate variability is not a measure of cardiac sympathetic tone but may be a measure of modulation of cardiac autonomic outflows by baroreflexes. *Exp. Physiol.* 96 1255–1261. 10.1113/expphysiol.2010.056259 21890520 PMC3224799

[B26] Greening S. G. Mitchell D. G. V. (2015). A network of amygdala connections predict individual differences in trait anxiety. *Hum. Brain Mapp.* 36 4819–4830. 10.1002/hbm.22952 26769550 PMC6869108

[B27] Gross J. J. (1998). The emerging field of emotion regulation: an integrative review. *Rev. Gen. Psychol.* 2 271–299. 10.1037/1089-2680.2.3.271

[B28] Guillen-Riquelme A. Buela-Casal G. (2011). Psychometric revision and differential item functioning in the State Trait Anxiety Inventory (STAI). *Psicothema* 23 510–515. 21774907

[B29] Harrison A. Sullivan S. Tchanturia K. Treasure J. (2010). Emotional functioning in eating disorders: attentional bias, emotion recognition and emotion regulation. *Psychol. Med.* 40 1887–1897. 10.1017/S0033291710000036 20102669

[B30] Houle M. S. Billman G. E. (1999). Low-frequency component of the heart rate variability spectrum: a poor marker of sympathetic activity. *Am. J. Physiol.* 276 H215–H223. 9887035 10.1152/ajpheart.1999.276.1.H215

[B31] Hu T. Zhang D. Wang J. Mistry R. Ran G. Wang X. (2014). Relation between emotion regulation and mental health: a meta-analysis review. *Psychol. Rep.* 114 341–362. 10.2466/03.20.PR0.114k22w4 24897894

[B32] Islam M. A. Steiger H. Jimenez-Murcia S. Israel M. Granero R. Agüera Z. (2015). Non-suicidal self-injury in different eating disorder types: relevance of personality traits and gender. *Eur. Eat. Disord. Rev.* 23 553–560. 10.1002/erv.2374 26075808

[B33] Jimenez-Murcia S. Fernandez-Aranda F. Kalapanidas E. Konstantas D. Ganchev T. Kocsis O. (2009). Playmancer project: a serious videogame as an additional therapy tool for eating and impulse control disorders. *Stud. Health Technol. Inform.* 144 163–166. 19592756

[B34] Keski-Rahkonen A. Mustelin L. (2016). Epidemiology of eating disorders in Europe: prevalence, incidence, comorbidity, course, consequences, and risk factors. *Curr. Opin. Psychiatry* 29 340–345. 10.1097/YCO.0000000000000278 27662598

[B35] Koenig J. Jarczok M. N. Warth M. Ellis R. J. Bach C. Hillecke T. K. (2014). Body mass index is related to autonomic nervous system activity as measured by heart rate variability–a replication using short term measurements. *J. Nutr. Health Aging* 18 300–302. 10.1007/s12603-014-0022-6 24626758 PMC12880227

[B36] Koenig J. Windham B. G. Ferrucci L. Sonntag D. Fischer J. E. Thayer J. F. (2015). Association strength of three adiposity measures with autonomic nervous system function in apparently healthy employees. *J. Nutr. Health Aging* 19 879–882. 10.1007/s12603-015-0508-x 26482688 PMC6121712

[B37] Kostro K. Lerman J. B. Attia E. (2014). The current status of suicide and self-injury in eating disorders: a narrative review. *J. Eat. Disord.* 2 19 10.1186/s40337-014-0019-x PMC4450853 26034603

[B38] Kreibig S. D. (2010). Autonomic nervous system activity in emotion: a review. *Biol. Psychol.* 84 394–421. 10.1016/j.biopsycho.2010.03.010 20371374

[B39] Lehrer P. M. Gevirtz R. (2014). Heart rate variability biofeedback: how and why does it work? *Front. Psychol.* 5:756 10.3389/fpsyg.2014.00756 PMC4104929 25101026

[B40] Linehan M. (1993). *Cognitive-Behavioral Treatment of Borderline Personality Disorder.* New York, NY: Guilford Press.

[B41] Malik M. Bigger J. T. Camm A. J. Kleiger R. E. Malliani A. Moss A. J. (1996). Heart rate variability. Standards of measurement, physiological interpretation, and clinical use. Task Force of the European Society of Cardiology and the North American Society of Pacing and Electrophysiology. *Circulation* 93 1043–1065. 10.1161/01.CIR.93.5.1043 8598068

[B42] Martinez-Azumendi O. Fernandez-Gomez C. Beitia-Fernandez M. (2001). Factorial variance of the SCL-90-R in a Spanish out-patient psychiatric sample] *Actas Esp. Psiquiatr.* 29 95–102. 11333527

[B43] Mazurak N. Enck P. Muth E. Teufel M. Zipfel S. (2011). Heart rate variability as a measure of cardiac autonomic function in anorexia nervosa: a review of the literature. *Eur. Eat. Disord. Rev.* 19 87–99. 10.1002/erv.1081 25363717

[B44] McKenzie K. C. Gross J. J. (2014). Nonsuicidal self-injury: an emotion regulation perspective. *Psychopathology* 47 207–219. 10.1159/00035809 24526099

[B45] Meule A. Freund R. Skirde A. K. Vögele C. Kübler A. (2012). Heart rate variability biofeedback reduces food cravings in high food cravers. *Appl. Psychophysiol. Biofeedback* 37 241–251. 10.1007/s10484-012-9197-y 22688890

[B46] Muehlenkamp J. J. Peat C. M. Claes L. Smits D. (2012). Self-injury and disordered eating: expressing emotion dysregulation through the body. *Suicide Life Threat. Behav.* 42 416–425. 10.1111/j.1943-278X.2012.00100.x 22646483

[B47] Nock M. K. Mendes W. B. (2008). Physiological arousal, distress tolerance, and social problem-solving deficits among adolescent self-injurers. *J. Consult. Clin. Psychol.* 76 28–38. 10.1037/0022-006X.76.1.28 18229980

[B48] Peschel S. K. V. Feeling N. R. Vögele C. Kaess M. Thayer J. F. Koenig J. (2016a). A meta-analysis on resting state high-frequency heart rate variability in Bulimia Nervosa. *Eur. Eat. Disord. Rev.* 24 355–365. 10.1002/erv.2454 27241070

[B49] Peschel S. K. V. Feeling N. R. Vögele C. Kaess M. Thayer J. F. Koenig J. (2016b). A systematic review on heart rate variability in Bulimia Nervosa. *Neurosci. Biobehav. Rev.* 63 78–97. 10.1016/j.neubiorev.2016.01.012 26828568

[B50] Pisetsky E. M. Haynos A. F. Lavender J. M. Crow S. J. Peterson C. B. (2017). Associations between emotion regulation difficulties, eating disorder symptoms, non-suicidal self-injury, and suicide attempts in a heterogeneous eating disorder sample. *Compr. Psychiatry* 73 143–150. 10.1016/j.comppsych.2016.11.012 27978502 PMC5263187

[B51] Ramírez E. Ortega A. R. Reyes Del Paso G. A. (2015). Anxiety, attention, and decision making: the moderating role of heart rate variability. *Int. J. Psychophysiol.* 490 490–496. 10.1016/j.ijpsycho.2015.10.007 26555079

[B52] Reyes del Paso G. A. Langewitz W. Mulder L. J. M. van Roon A. Duschek S. (2013). The utility of low frequency heart rate variability as an index of sympathetic cardiac tone: a review with emphasis on a reanalysis of previous studies. *Psychophysiology* 50 477–487. 10.1111/psyp.12027 23445494

[B53] Rodas G. Pedret Carballido C. Ramos J. Capdevila L. (2008). Variabilidad de la frecuencia cardíaca: concepto, medidas y relación con aspectos clínicos (I). *Arch. Med. Deport.* 25 41–48.

[B54] Sakakibara R. Kitahara M. (2016). [The relationship between cognitive emotion regulation questionnaire (CERQ) and depression, anxiety: meta-analysis]. *Shinrigaku Kenkyu* 87 179–185. 10.4992/jjpsy.87.15302 27476268

[B55] Salman S. Idrees J. Hassan F. Idrees F. Arifullah M. Badshah S. (2014). Predictive factors of suicide attempt and non-suicidal self-harm in emergency department. *Emergency* 2 166–169. 26495374 PMC4614564

[B56] Scolnick B. Mostofsky D. I. Keane R. J. (2014). Pilot study employing heart rate variability biofeedback training to decrease anxiety in patients with eating disorders. *J. Eat. Disord.* 2:17 10.1186/2050-2974-2-17 PMC4050420 24917934

[B57] Selby E. A. Bender T. W. Gordon K. H. Nock M. K. Joiner T. E. (2012). Non-suicidal self-injury (NSSI) disorder: a preliminary study. *Personal. Disord.* 3 167–175. 10.1037/a0024405 22452757

[B58] Selby E. A. Smith A. R. Bulik C. M. Olmsted M. P. Thornton L. McFarlane T. L. (2010). Habitual starvation and provocative behaviors: two potential routes to extreme suicidal behavior in anorexia nervosa. *Behav. Res. Ther.* 48 634–645. 10.1016/j.brat.2010.03.016 20398895 PMC4731222

[B59] Siepmann M. Aykac V. Unterdörfer J. Petrowski K. Mueck-Weymann M. (2008). A pilot study on the effects of heart rate variability biofeedback in patients with depression and in healthy subjects. *Appl. Psychophysiol. Biofeedback* 33 195–201. 10.1007/s10484-008-9064-z 18807175

[B60] Solano R. Fernández-Aranda F. Aitken A. López C. Vallejo J. (2005). Self-injurious behaviour in people with eating disorders. *Eur. Eat. Disord. Rev.* 13 3–10. 10.1002/erv.618

[B61] Solano Pinto N. Cano Vindel A. (2012). Anxiety in eating disorders: a comparative study. *Psicothema* 24 384–389. 22748728

[B62] Spielberger C. D. Gorsuch R. L. Cubero N. S. Lushene R. E. (1982). *STAI, Cuestionario de Ansiedad Estado-rasgo: Manual.* Madrid: TEA Ediciones, S.A.

[B63] Spielberger C. D. Gorsuch R. L. Lushene R. E. (1970). *Manual for the State-Trait Anxiety Inventory.* Palo Alto, CA: Consulting psychologists Press.

[B64] Stein D. Lilenfeld L. R. Wildman P. C. Marcus M. D. (2004). Attempted suicide and self-injury in patients diagnosed with eating disorders. *Compr. Psychiatry* 45 447–451. 10.1016/j.comppsych.2004.07.011 15526255

[B65] Svaldi J. Griepenstroh J. Tuschen-Caffier B. Ehring T. (2012). Emotion regulation deficits in eating disorders: a marker of eating pathology or general psychopathology? *Psychiatry Res.* 197 103–111. 10.1016/j.psychres.2011.11.009 22401969

[B66] Thayer J. F. Ahs F. Fredrikson M. Sollers J. J. Wager T. D. (2012). A meta-analysis of heart rate variability and neuroimaging studies: implications for heart rate variability as a marker of stress and health. *Neurosci. Biobehav. Rev.* 36 747–756. 10.1016/j.neubiorev.2011.11.009 22178086

[B67] Tuck N. L. Grant R. C. I. Sollers J. J. Booth R. J. Consedine N. S. (2016). Higher resting heart rate variability predicts skill in expressing some emotions. *Psychophysiology* 53 1852–1857. 10.1111/psyp.12755 27565951

[B68] Vansteelandt K. Claes L. Muehlenkamp J. De Cuyper K. Lemmens J. Probst M. (2013). Variability in affective activation predicts non-suicidal self-injury in eating disorders. *Eur. Eat. Disord. Rev.* 21 143–147. 10.1002/erv.2220 23239050

[B69] Visted E. Sørensen L. Osnes B. Svendsen J. L. Binder P.-E. Schanche E. (2017). The association between self-reported difficulties in emotion regulation and heart rate variability: the salient role of not accepting negative emotions. *Front. Psychol.* 8:328 10.3389/fpsyg.2017.00328 PMC5343522 28337160

[B70] Weinberg A. Klonsky E. D. Hajcak G. (2009). Autonomic impairment in borderline personality disorder: a laboratory investigation. *Brain Cogn.* 71 279–286. 10.1016/j.bandc.2009.07.014 19751961

[B71] Williams D. P. Cash C. Rankin C. Bernardi A. Koenig J. Thayer J. F. (2015). Resting heart rate variability predicts self-reported difficulties in emotion regulation: a focus on different facets of emotion regulation. *Front. Psychol.* 6:261 10.3389/fpsyg.2015.00261 PMC4354240 25806017

[B72] You J. Deng B. Lin M.-P. Leung F. (2016). The interactive effects of impulsivity and negative emotions on adolescent nonsuicidal self-injury: a latent growth curve analysis. *Suicide Life Threat. Behav.* 46 266–283. 10.1111/sltb.12192 26436464

